# Associations Between Smoking Abstinence, Inhibitory Control, and Smoking Behavior: An fMRI Study

**DOI:** 10.3389/fpsyt.2021.592443

**Published:** 2021-04-01

**Authors:** Spencer Bell, Brett Froeliger

**Affiliations:** ^1^Department of Neuroscience, Medical University of South Carolina, Charleston, SC, United States; ^2^Psychology Department, Dixie State University, St. George, UT, United States; ^3^Department of Psychiatry, University of Missouri, Columbia, MI, United States; ^4^Department of Psychological Sciences, University of Missouri, Columbia, MI, United States

**Keywords:** inhibitory control, smoking, withdrawal, fMRI, relapse

## Abstract

Nicotine addiction is associated with dysregulated inhibitory control (IC), mediated by corticothalamic circuitry including the right inferior frontal gyrus (rIFG). Among sated smokers, worse IC task performance and greater IC-related rIFG activity have been shown to be associated with greater relapse vulnerability. The present study investigated the effects of smoking abstinence on associations between IC task performance, rIFG activation, and smoking behavior. Smokers (*N* = 26, 15 female) completed an IC task (Go/Go/No-go) during fMRI scanning followed by a laboratory-based smoking relapse analog task (SRT) on two visits: once when sated and once following 24 h of smoking abstinence. During the SRT, smokers were provided with monetary rewards for incrementally delaying smoking. A significant main effect of No-go accuracy on latency to smoke during the SRT was observed when collapsing across smoking states (abstinent vs. sated). Similarly, a significant main effect of IC-related activation in rIFG on SRT performance was observed across states. The main effect of state, however, was non-significant in both of these models. Furthermore, the interaction between smoking state and No-go accuracy on SRT performance was non-significant, indicating a similar relationship between IC and lapse vulnerability under both sated and abstinent conditions. The state X rIFG activation interaction on SRT performance was likewise non-significant. *Post-hoc* whole brain analyses indicated that abstinence resulted in greater IC-related activity in the right middle frontal gyrus (MFG) and insula. Activation during IC in these regions was significantly associated with decreased No-go accuracy. Moreover, greater abstinence induced activity in right MFG during IC was associated with smoking sooner on the SRT. These findings are bolstered by the extant literature on the effects of nicotine on executive function and also contribute novel insights on how individual differences in behavioral and neuroimaging measures of IC may influence relapse propensity independent of smoking state.

## Introduction

Nicotine addiction is associated with dysregulated prefrontal-mediated executive function including multiple forms of memory (1–3), reward processing (4–7), emotion-cognition interactions (8–11), and inhibitory control (IC) (12–15). Inhibitory control is defined as the ability to withhold a prepotent response in favor of performing context-relevant, goal-directed behavior (16). Stimulus-driven, context-dependent IC is carried out through a corticothalamic network that includes the right inferior frontal gyrus (rIFG), pre-supplementary motor area (preSMA), thalamus, and subthalamic nucleus (STN) (17–19). Numerous studies indicate that nicotine withdrawal induces deficits on performance of executive function tasks (20–25), including IC tasks (15). Notably, nicotine withdrawal-induced disruption of executive function may represent a significant factor underlying smoking maintenance and relapse (26). Previous research has also shown that measures of executive function (Stroop task performance) predict the ability to delay smoking in a laboratory setting under conditions of abstinence (27). The predictive relationship, however, between the mechanisms underlying withdrawal-induced cognitive deficits and relapse outcomes remain poorly understood. A better understanding of the factors and mechanisms undergirding relapse propensity may facilitate individually tailored treatments and improve their effectiveness.

Worse performance on IC tasks has been shown to be predictive of smoking relapse in both real-world (28) and laboratory settings (29). Moreover, among smokers at baseline (i.e., pre-quit), IC task-based functional connectivity in corticothalamic circuitry mediates the association between IC task performance and smoking behavior—both in laboratory and naturalistic contexts (29). Despite current evidence to suggest that baseline IC predicts relapse vulnerability, the effects of abstinence on this relationship remain largely uncharacterized. While our previous research has characterized the predictive relationship between the behavioral and neural correlates of IC and resisting smoking during a laboratory analog of relapse, the primary goal of this study was to investigate the effects of smoking abstinence on IC and smoking behavior. Consistent with previous research (15), we hypothesized that abstinence, as compared to satiety, would result in greater IC-related activity in rIFG and worse behavioral performance. We further hypothesized that rIFG activation and IC task performance would be associated with heightened lapse vulnerability, as measured by a smoking relapse analog task [SRT; (29)].

## Materials and Methods

### Participants and Timeline

The study was approved by the institutional review board at the Medical University of South Carolina. Participants gave written informed consent and received financial compensation. Participants (*n* = 30) were recruited via flyers and internet advertisements. Participants were right-handed smokers 18+ yrs. of age, smoking for ≥2 years, >10 cigarettes/day with an expired CO concentration of ≥10 ppm. Participants were not currently seeking treatment for smoking. Exclusion criteria were: lifetime history of an Axis 1 disorder, suicidality, or diagnosis of a substance use disorder (other than nicotine) as assessed with the MINI (30); any physical or mental disability affecting completion of assessments; use of psychotropic and antiepileptic medications in the last month; positive urine drug screen (including marijuana); positive pregnancy test; presence of an unstable medical illness; current or past psychosis; history of major neurological illness or head injury resulting in loss of consciousness; any contraindication to MRI; and any other condition that would impact participant safety/compliance or confound interpretation of study results.

Following informed consent, screening, and a training visit, participants performed a well-validated IC task (31) during the collection of blood oxygen-level dependent (BOLD) signal during fMRI. Scanning occurred on two separate visits, with an average of 4.77 days elapsed between each (maximum days = 15, minimum days = 2). On a designated “Sated” visit, participants smoked as usual and also smoked one cigarette 30 min prior to scanning. On a designated “Abstinent” visit, smokers maintained smoking abstinence for 24 h prior to the experimental session. Smoking compliance with each visit was verified through expired CO. The order of these two conditions was counterbalanced. To investigate whether behavioral differences between conditions could be due to practice effects on the IC-task, we performed a Mann-Whitney *U*-test on the distributions of days elapsed between scanning sessions, comparing those subjects who completed the Abstinent condition first (*n* = 13, median days elapsed = 2) with those who completed it second (*n* = 13, median days elapsed = 4). These distributions were not significantly different (*p* = 0.418), leading us to conclude that any practice effects were controlled for by counterbalancing. Scanning was followed by the SRT (29) designed to measure smoking relapse propensity. Four participants were excluded due to poor performance on the IC task (<75% Go trials correct, as described below), thus the final sample was *N* = 26 (15 females, 11 males). Data collected on this sample of participants during the Sated visit has been previously reported (29). This report is the first, however, to report on data collected during the Abstinent visit. One subject's SRT data was not recorded on the Abstinent visit due to technical problems, therefore analyses including this measure were done on a sample of *N* = 25.

### Behavioral, Biomarker, and Self-Report Measures

#### Smoking History, Nicotine Dependence, and State Measures

Smoking history was reported using a general questionnaire to assess duration and frequency of smoking. Nicotine dependence was assessed using the Fagerström Test of Nicotine Dependence (32). The Shiffman/Jarvik Withdrawal scale (33) was administered at each experimental visit to assess nicotine withdrawal.

#### Tobacco Use Biomarker

Expired air CO concentrations were measured using a handheld CO monitor (Vitalograph, Lenexa, KS) to establish eligibility and baseline level of smoking and repeated prior to each experimental session to evaluate compliance with each condition (Sated > 10 ppm; Abstinent < 6 ppm).

#### Inhibitory Control Task

The IC task used in the study was the Go/Go/No-Go task (31) involving randomly presented colored circles in 3 types of trials: frequent gray (Go, 75.4%; *n* = 388 trials), rare yellow (Rare Go, 12.3%; *n* = 65 trials), and rare blue (No Go, 12.3%; *n* = 65 trials). Participants were instructed to respond as quickly as possible to “Go” and “Rare Go” trials by pressing a button with their right index finger and to withhold any response to “No Go” trials. Each event of interest was presented for 400 ms, separated by a 400 ms blank screen (ISI = 800 ms); spacing between rare trials (Rare Go or No Go) ranged from 8.8 to 12.0 s over the course of the task (duration = 7.22 min). The primary outcome of interest used as a measure of IC was the percent of No Go trials correct. In order to control for lapses in attention, the percentage of No Go correct trials was adjusted by omitting trials in which a subject failed to respond to a “Go” trial immediately preceding the No Go trial. See Supplementary Figure 1 for an overview of the Go/Go/No-Go task.

#### Smoking Relapse Analog Task (SRT)

In order to assess smokers' ability to resist smoking, we assessed smoking behavior in the context of a monetary contingency to maintain abstinence. Following their fMRI scanning session, participants performed a picture-viewing task (5, 29) consisting of images from the International Affective Picture System. Participants were instructed to provide self-reported mood ratings (1—most negative to 8—most positive) in response to each image. The images were presented for 10 s each with a 5-s interval after each image in which mood ratings were collected and a 3- to 7-s rest interval preceding each image. The task consisted of 6-min blocks, each with a mixture of negative, neutral, and positive images. Participants received $1 for each 6-min period that they completed the task and did not smoke. At any point following a 6-min task block participants were allowed to choose either to stop the task and smoke a cigarette or to continue performing the task for more money. The maximum number of 6-min blocks a participant was permitted to complete was 10. The primary objective of the picture viewing component of the task was to keep participants engaged in an activity to reduce boredom and the mood ratings were not analyzed as part of the study presented herein.

#### MRI Data

Data were collected on a Magnetom TrioTim 3TMR scanner (Siemens, Erlangen, Germany) with a 32-channel head coil. *T1-weighted structural:* A high-resolution anatomical scan (magnetization prepared rapid gradient echo) was acquired, to allow subsequent registration to functional images and region-of-interest (ROI) definition [parameters: repetition/echo time (TR/TE) = 1,900/2.26 ms; flip angle (FA) = 9°; field of view (FOV) = 256 mm^2^; voxel size = 1 mm^2^; 192 contiguous 1-mm-thick slices. *Functional BOLD imaging:* An EP2D-BOLD sequence was performed to acquire functional activity during the IC task with the following parameters: TR/TE = 2,000/30 ms; FA = 90°; 36 sections; and voxel size, 3.3 × 3.3 × 3.0 mm.

### Data Analysis Strategy

#### Behavioral Data

Self-report measures and behavioral variables were analyzed in SPSS and R with a statistical threshold of α = 0.05. Distributions of variables, including the distributions of residuals in bivariate data, were examined for normality violations using the Shapiro-Wilk test. Homoscedasticity of bivariate data was also examined using the Koenker test. When violations of normality and/or heteroscedasticity were found, non-parametric tests were employed. Matched pairs *t*-tests and Wilcoxon Signed Ranks Tests were used to compare Shiffman/Jarvik Withdrawal subscales between states. A state (two levels) by trial type (three levels) repeated-measures ANOVA was used to examine IC task performance and a Mann Whitney *U*-Test was employed to examine state effects on SRT performance. Cox proportional hazard regression analysis of survival curves in SPSS was used to examine relationships between No Go Accuracy and SRT performance under both Sated and Abstinent conditions. A third Cox proportional hazard regression model was employed in R using the coxph function with state (Abstinent vs. Sated) and No Go Accuracy as terms in the model as well as a state by No Go Accuracy interaction term to examine the effects of smoking state on the relationship between IC task performance and SRT performance. Pearson and Spearman correlation tests were employed to examine relationships between No Go Accuracy and brain activation in exploratory *post-hoc* analyses, as described subsequently.

#### fMRI Data

**Figure 1:**
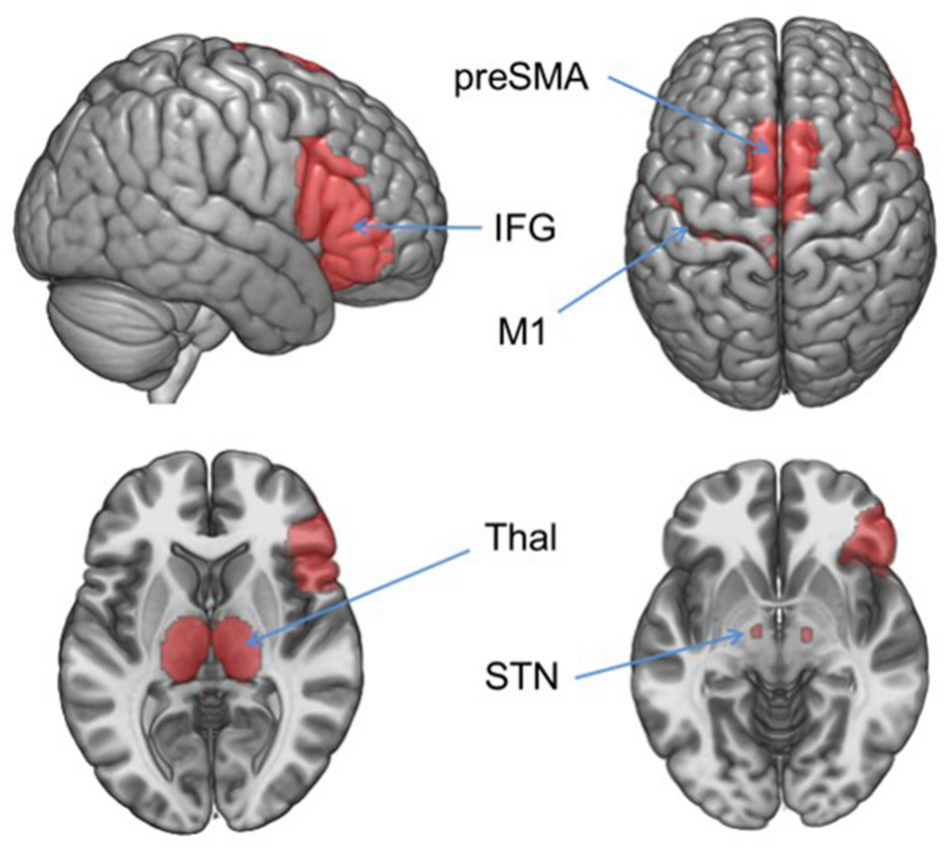
Inhibitory control network mask. Hypothesis-driven fMRI analyses were restricted to the regions in this mask which included right inferior frontal gyrus (IFG), left precentral gyrus (M1), and bilateral thalamus (Thal), subthalamic nucleus (STN), and pre-supplementary motor area (preSMA). Areas were defined in WFU Pickatlas.

Functional data were preprocessed using SPM12 which included correction for slice acquisition time; motion correction using a rigid-body rotation and translation algorithm (34); motion outlier detection [framewise displacement >4 mm (~1 acquisition voxel) http://www.nitrc.org/projects/artifact_detect] and correction (via nearest-neighbor interpolation); despiking at 4% of global mean (http://cibsr.stanford.edu/tools/human-brain-project/artrepair-software.html); temporal realignment using B-spline interpolation; normalization into standard stereotaxic space (MNI) with a 1.5 mm^3^ voxel size; and smoothing with a 10 mm FWHM Gaussian filter. *Analysis:* For the IC task, each participant's data from each session were entered into a first-level, whole-brain analysis using the General Linear Model (35) to examine BOLD response to each of the 5 trial types: Correct No Go (successful inhibition), Incorrect No Go (error of commission), Correct Rare Go (novel-target detection), Incorrect Rare Go (novel-target error of omission), and Incorrect Go (error of omission). Each event type was modeled as a delta regressor at the onset of the event and convolved with a canonical hemodynamic response function. Motion parameters were included as covariates. A high-pass filter (128 s; 0.008 Hz) was applied to remove slow signal drift. Hypothesis testing was conducted within an explicit IC network mask defined in WFU Pickatlas (http://fmri.wfubmc.edu/software/pickatlas) including rIFG, thalamus, subthalamic nucleus, preSMA, and motor cortex [(19); Figure 1].

To investigate the relationship between activation in the rIFG and behavioral variables, we first examined the main effect of trial type in a state (2 levels: Sated, Abstinent) by trial type (2 levels: No Go Correct, Rare Go Correct) repeated measures ANOVA within the IC network mask. This analysis revealed a large cluster of activation in rIFG (*F* = 59.56, k_E_ = 2,169, *p* < 0.0001). In order to further refine search area for a functional ROI with greater anatomical specificity, one-way *F*-tests were conducted on Sated and Abstinent visits alone to examine the effects of the task (No Go Correct vs. Rare Go Correct BOLD signal) on each visit at a significance threshold of *p* < 0.05, FWE-corrected at the voxel level. A conjunction analysis was then performed on these two models to determine the overlap of IC-Activation during Sated and Abstinent visits. As outlined in Results, this conjunction analysis revealed three adjacent clusters in rIFG, one of which was selected for further analysis as it demonstrated overlap with the rIFG cluster previously found to be associated with SRT outcomes (29). Brain activation between all three clusters during No Go trials was also significantly correlated (all *p*'s < 0.04). Upon selection of this cluster, mean percent signal change (PSC) was extracted using MarsBaR (http://marsbar.sourceforge.net/) and subsequently examined in SPSS. Mean PSC associated with Rare Go Correct trials was subtracted from No Go Correct mean PSC in SPSS to control for novelty detection to a rare target. This No Go Correct—Rare Go Correct contrast is referred to henceforth as IC-Activation. Wilcoxon signed-rank tests compared mean IC-Activation response on Abstinent and Sated visits. The relationship between IC-Activation response from these ROIs and SRT performance, as well as the interaction between smoking state and this relationship, was examined using Cox proportional hazard regression analysis of survival curves using the same analysis strategy as detailed previously for No Go Accuracy.

### Exploratory *Post-hoc* Analyses

To further investigate the effects of IC on BOLD response during abstinence, a one-way ANOVA was employed in SPM to examine main effects of trial type (No Go Correct, Rare Go Correct) on whole-brain activity during the IC task in the Abstinent condition using a threshold of *p* < 0.05, FWE corrected at the voxel level. Percent signal change was extracted from clusters where a significant main effect was found in this contrast. Relationships with behavioral outcomes were then examined in SPSS. The criteria for selection of the functional ROIs were independent of the actual behavioral outcomes examined (No Go accuracy and time to smoke during the SRT), thus avoiding bias due to circular analysis (36).

## Results

### Demographic Characteristics and Self-Report Measures

Demographic characteristics of the sample are detailed in Table 1. Except for somatic symptoms and arousal, all self-reported withdrawal symptoms were significantly higher under the Abstinent, as compared to the Sated condition (all *p*'s < 0.04; Table 2). The expired CO values differed significantly between states (*t* = 6.837, *p* < 0.0001, Table 2).

**Table 1:** Subject demographics and baseline self–report measures.

	**Overall sample**
Total *N*	26
Age	34.9 (10.3)
Sex distribution	15 F, 11 M
Years of education	14.1 (2.0)
**Race distribution**	
Black or African American	10
White	14
Asian	2
**Baseline clinical measures**	
Nicotine dependence (FTND)	4.5 (1.7)
Years smoking	15.4 (8.3)
Average daily cigarettes	14.8 (4.4)
Pack-years	11.8 (8.0)

*Mean (SD) indicated where applicable*.

**Table 2:** State measures: nicotine withdrawal and expired CO.

***Shiffman-Jarvik Withdrawal Subscales***	**Sated**	**Abstinent**	***p***
Craving	2.6 (0.19)	5.0 (0.28)	***<0.0001***
Negative affect	3.0 (0.13)	3.9 (0.13)	***<0.0001***
Appetite disturbance	1.2 (0.07)	1.8 (0.17)	**<0.0001**
Arousal	6.1 (0.15)	5.6 (0.21)	***0.03***
Somatic symptoms	1.2 (0.05)	1.2 (0.06)	0.876
Habit withdrawal	1.3 (0.11)	2.5 (0.28)	**<0.0001**
***Expired CO (ppm)***	4.3 (0.67)	23.5 (3.14)	***<0.0001***

*Standard error (SE) listed next to mean in parentheses. Italics, Matched pairs T-test, Non-italics, Wilcoxon Signed Ranks Test. Bold p-values are significant at α = 0.05*.

### Behavioral Task Results

#### Inhibitory Control Task Behavioral Performance

A significant main effect of trial type on accuracy was shown [*F*_(1.05, 26.35)_ = 127.70 (Greenhouse-Geisser Corrected), *p* < 0.0001, partial η^2^ = 0.836], indicating worse accuracy on No Go vs. Go and Rare Go trials, but no significant main effect of state [*F*_(1, 25)_ = 1.84, *p* = 0.187] or state by trial type interaction [*F*_(1.07, 26.66)_ = 0.424 (Greenhouse-Geisser Corrected), *p* = 0.533]. When examined alone *post-hoc*, average No Go accuracy did not significantly differ between Abstinent and Sated states (*t* = 0.975, *p* = 0.339) (Table 3). Additionally, neither Go nor Rare Go trial accuracy differed significantly between states (*t* = 1.29, *p* = 0.210; *t* = 0.28, *p* = 0.780; Table 3).

**Table 3:** Go/Go/No-Go task and smoking relapse-analog task performance.

	**Sated**	**Abstinent**	**Delta**	***p***
**Go/Go/No-Go task trial types**	**Mean % correct (SE)**	**Mean % correct (SE)**	**Mean change (SE)**	
No Go	59.41 (3.81)	56.67 (3.19)	−2.75 (2.81)	0.339
Go	93.23 (0.97)	92.05 (1.22)	−0.35 (1.23)	0.210
Rare Go	93.13 (1.30)	92.79 (1.45)	−1.18 (0.92)	0.780
**SRT**	**Median (IQR)**	**Median (IQR)**	**Median change (IQR)**	
Time to smoke (min)	48 (42)	12 (54)	0.0 (18)	**0.019**

*Italics, Matched pairs T-test; non-italics, Mann-Whitney U-test. Bolded p-values are significant at α = 0.05*.

#### Smoking Relapse Analog Task (SRT) Performance

Participants engaged in smoking during the SRT significantly sooner [*Z* = −2.281, *p* = 0.019 (Exact)] on the Abstinent visit as compared to the Sated visit (Table 3).

#### Associations Between IC Task Performance and SRT Performance

**Figure 2:**
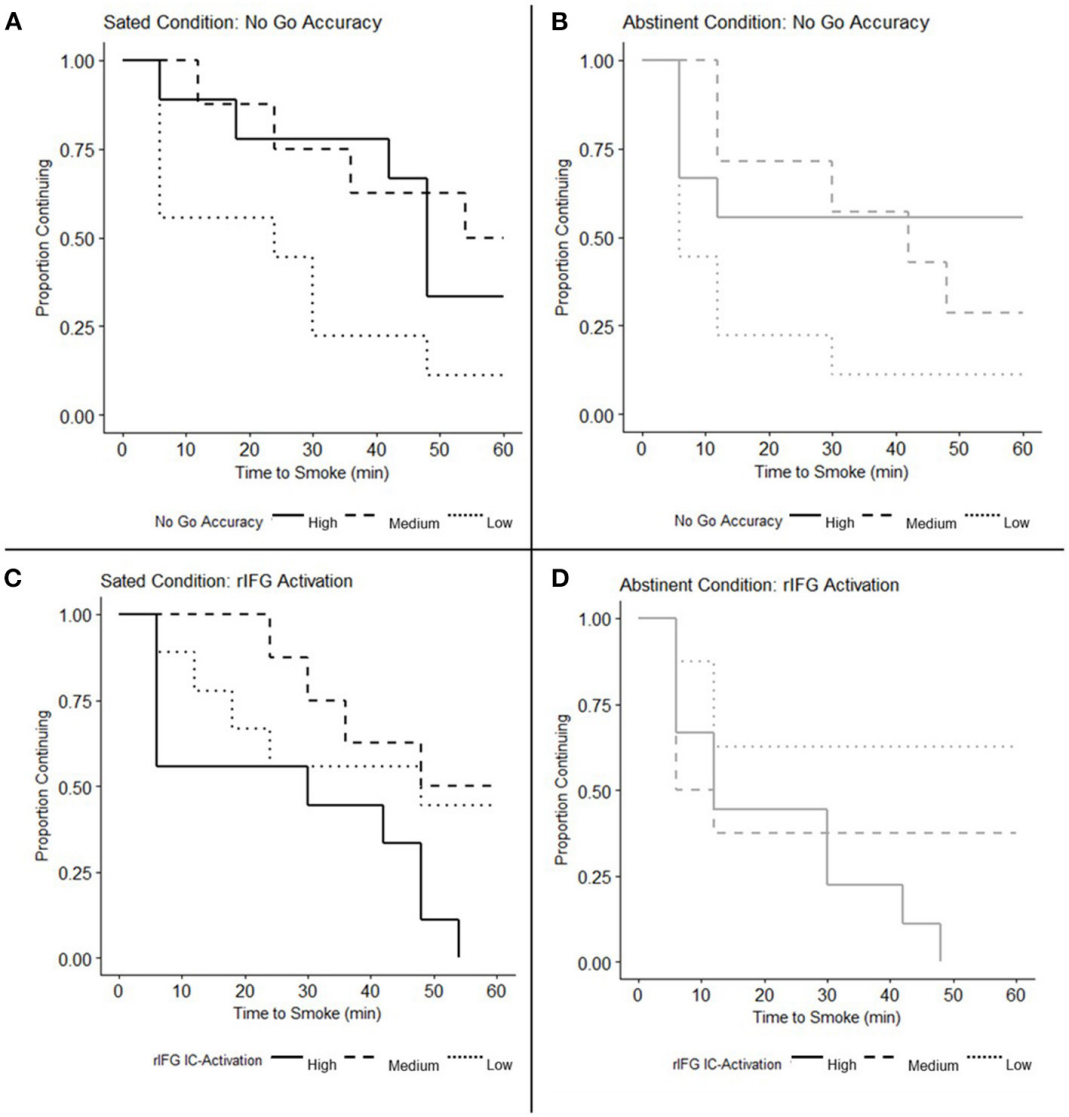
No Go accuracy and inhibitory control (IC) activation in right inferior frontal gyrus (rIFG) related to smoking relapse analog task (SRT) performance. **(A,B)** Survival curves depicting Time to Smoke on the SRT under the **(A)** Sated condition (*N* = 26) and **(B)** Abstinent condition (*N* = 25) are stratified by participants with high, medium, and low No Go accuracy for illustrative purposes. Cox proportional hazards regression models reveal a significant main effect of No Go accuracy when collapsing across states (*p* = 0.006), but indicate that No Go accuracy does not significantly predict time to smoke when examined under the Sated (*p* = 0.083) and Abstinent conditions (*p* = 0.057) alone. The main effect of state on SRT performance was non-significant (*p* = 0.23) as was the interaction between No Go Accuracy and smoking state (*p* = 0.25), indicating a similar relationship between No Go Accuracy and SRT performance across both states. **(C,D)** Survival curves depicting Time to Smoke on the SRT under the **(C)** Sated and **(D)** Abstinent conditions are stratified by participants with high, medium, and low rIFG IC-Activation for illustrative purposes. Cox proportional hazards regression models indicate that the main effect of rIFG IC-Activation on SRT performance is significant when collapsing across states (*p* = 0.025); when examined alone, rIFG IC-Activation predicts time to smoke on the Sated (*p* = 0.019), but not Abstinent visit (*p* = 0.135). The main effect of smoking state on the relationship between rIFG IC-Activation and SRT performance was non-significant (*p* = 0.61) as well as the interaction between rIFG IC-Activation and smoking state (*p* = 0.47).

The interaction between smoking state and No Go accuracy on SRT performance was non-significant in the Cox proportional hazard regression model [*B* = 0.017, Hazard Ratio (95% CI) = 1.017 (0.989, 1.046), *p* = 0.25], indicating a similar relationship between No Go accuracy and SRT performance under both Sated and Abstinent conditions. The main effect of smoking state on SRT performance was also non-significant [*B* = −1.042, Hazard Ratio (95% CI) = 0.353 (0.065, 1.904), *p* = 0.23] while the main effect of No Go accuracy was significant when collapsing across states [*B* = −0.036, Hazard Ratio (95% CI) = 0.965 (0.940, 0.990), *p* = 0.006] indicating that greater No Go accuracy predicted greater smoking latency. Subsequent Spearman correlation analysis revealed that change in No Go Accuracy due to state (Abstinent — Sated) was not significantly related to change in SRT performance (ρ = −0.252, *p* = 0.224). Although the relationship between No Go accuracy and time to smoke during the SRT during the Sated visit (when examined alone) was similar in direction to previous findings (29), this relationship was non-significant in the Cox proportional hazard model [*B* = −0.021, Hazard Ratio (95% CI) = 0.979 (0.956, 1.003), *p* = 0.083, Figure 2A]. This pattern was also evident during the Abstinent visit [*B* = −0.028, Hazard Ratio (95% CI) = 0.972 (0.944, 1.001), *p* = 0.057, Figure 2B]. Figures 2A,B depict survival curves stratified by high (*n* = 9), medium (*n* = 8), and low (*n* = 9) No Go accuracy for illustrative purposes (i.e., the data was not subdivided for analysis but only to illustrate differential patterns in survival rates based on No Go accuracy).

### Inhibitory Control Task fMRI Results

#### Main Effect of IC Task on BOLD Response

Within the inhibitory control network mask (see Methods and Figure 1) a main effect of trial type on BOLD response was found in the rIFG and preSMA during both Sated and Abstinent visits, with activation greater during No Go Correct than Rare Go Correct trials (Supplementary Table 1 and Supplementary Figure 2). The three significant clusters resulting from this conjunction analysis between these regions across states are shown in Supplementary Figure 2. As described in Materials and Methods, percent signal change used in the IC-Activation contrast was extracted from the largest of these ROIs (MNI: 51,12,16, k_E_ = 219 mm^3^, *p* = 0.006, FWE-corrected) to examine relationships with behavior.

#### Main Effect of Smoking State on Right Inferior Frontal Gyrus Activation

No significant main effect of smoking state was found on IC-Activation within the rIFG previously identified in the conjunction analysis (*z* = −0.521, *p* = 0.603).

#### Associations Between IC-Activation and SRT Performance

The interaction between smoking state and IC-Activation in the rIFG on SRT performance was non-significant [*B* = 1.127, Hazard Ratio (95% CI) = 3.088 (0.143, 66.423), *p* = 0.47], indicating no significant difference in the relationship between rIFG activity and SRT performance in abstinent vs. sated smokers. The main effect of smoking state on the relationship between IC-Activation and SRT performance was also non-significant [*B* = −0.179, Hazard Ratio (95% CI) = 0.836 (0.421, 1.662), *p* = 0.610]. The main effect of rIFG IC-Activation was significant, however, when collapsing across states [*B* = 2.990, Hazard Ratio (95% CI) = 19.881 (1.464, 269.982), *p* = 0.025], indicating that greater IC-Activation was associated with smoking sooner during the SRT. Spearman correlation analysis revealed that state-related change in rIFG IC-Activation (Abstinent – Sated) was not significantly related to change in SRT performance (ρ = 2.71, *p* = 0.189). Cox regression analyses indicted that greater IC-Activation in the rIFG was associated with shorter time to smoke during the SRT on the Sated visit, when examined alone [*B* = 4.87, Hazard Ratio (95% CI) = 130.08 (2.22, 7610.99), *p* = 0.019, Figure 2C]. This relationship was non-significant under the Abstinent condition, although in the same direction as in the Sated visit [*B* = 2.416, Hazard Ratio (95% CI) = 11.204 (0.47, 267.00), *p* = 0.135, Figure 2D].

### Exploratory *Post-hoc* Analyses of Smoking Abstinence

#### Main Effect of IC Task on Whole-Brain BOLD Response

**Figure 3:**
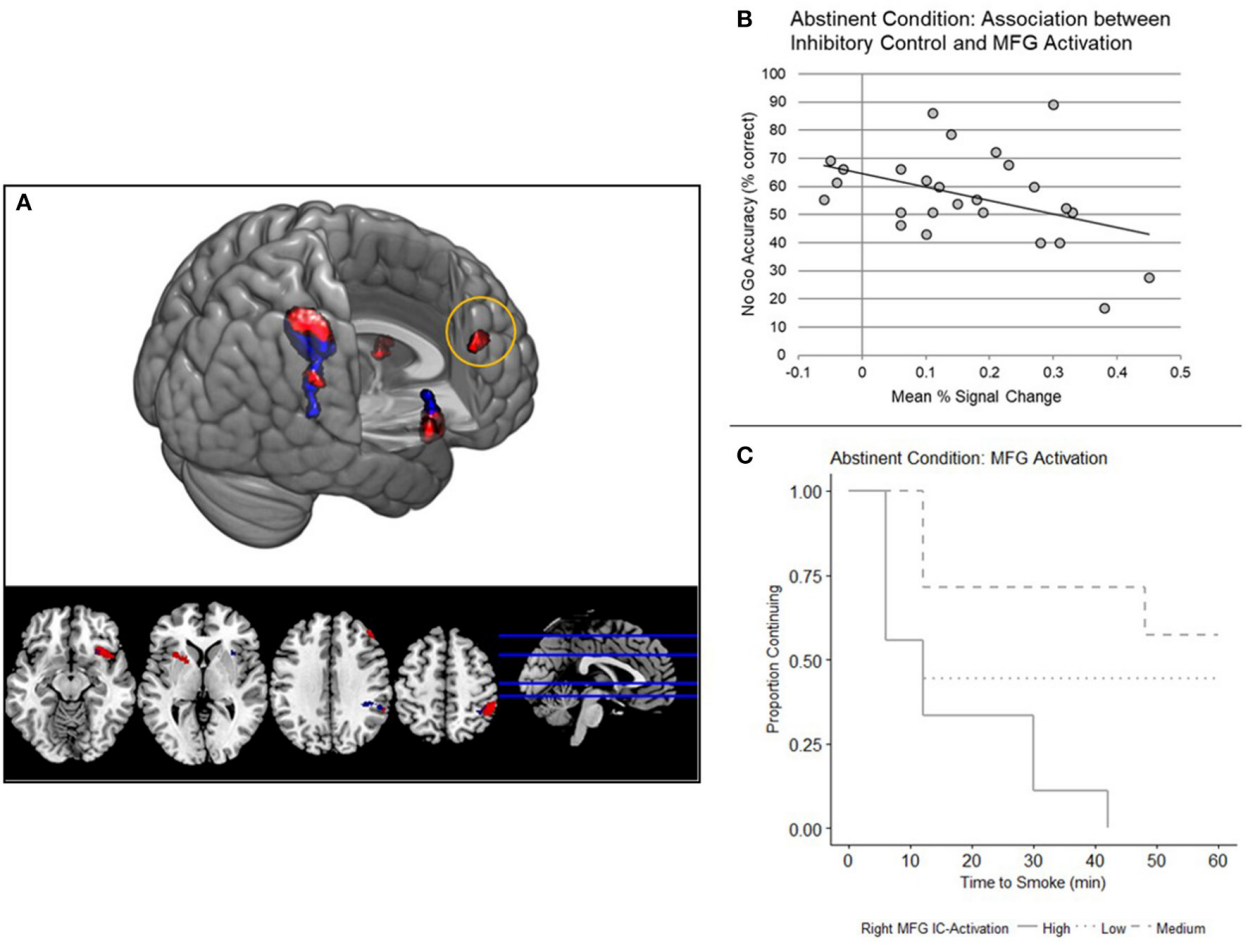
**(A)** Whole brain analyses: main effect of inhibitory control (IC) task. Main effect of trial type (No Go Correct, Rare Go Correct) across the whole brain, examined independently in a one-way ANOVA under each condition (red, Abstinent; blue, Sated; *N* = 26, *p* < 0.05 FWE corrected). **(B)** Right middle frontal gyrus (MFG) IC-Activation and No Go Accuracy. IC-Activation (Mean PSC) in right MFG is negatively correlated with No Go accuracy (*N* = 26, *p* = 0.040). Mean percent signal change is from the No Go Correct - Rare Go Correct contrast. **(C)** Right MFG IC-Activation and smoking relapse analog task (SRT) Performance. Survival curves were stratified by high, medium, and low MFG IC-Activation for illustrative purposes. A Cox regression model indicated that right MFG IC-Activation predicts time to smoke during the SRT (*N* = 25, *p* = 0.038).

In the Abstinent condition, a significant main effect of trial type (p < 0.05 FWE) on BOLD response was shown in the right insula, right parietal cortex, right middle frontal gyrus (MFG), and left putamen (Supplementary Table 2 and Figure 3A).

#### Associations Between Whole-Brain IC-Activation and Behavioral Outcomes

IC-Activation in the right middle frontal gyrus (MFG) was negatively associated with No Go accuracy and latency to smoke under the Abstinent condition (Table 4 and Figures 3B,C). No Go accuracy was also negatively associated with IC-Activation in right insula (Table 4). To ascertain whether these relationships also existed under the Sated condition, we extracted percent signal change data collected under the Sated condition from the aforementioned MFG and Insula clusters. The relationships between behavioral variables (latency to smoke during SRT and No Go Accuracy) in MFG and Insula were non-significant under the Sated condition (*p* > 0.17).

**Table 4:** Relationship between abstinent IC-Activation and behavioral variables (whole brain analyses).

			**Relationship with behavior**			
			**No Go accuracy**		**Time to smoke**	
**Region**	**MNI**	**Mean PSC** **(No Go – Rare Go)**	**Correlation coefficient**	***p***	**Cox proportional** **Hazard Ratio**	***p***
R. Insula	34,18,−12	0.15 (0.027)	–*0.448*	***0.022***	10.8	0.204
R. Parietal Cortex	52, −40,54	0.18 (0.032)	−0.111	0.590	4.5	0.337
R. MFG	44,39,33	0.16 (0.027)	–*0.405*	***0.040***	57.0	**0.038**
L. Putamen	−20,9,2	0.11 (0.020)	–*0.064*	*0.756*	1.8	0.829
R. Parietal Cortex	60, −44,30	0.13 (0.027)	–*0.240*	*0.238*	17.2	0.172

*Standard error (SE) listed next to mean in parentheses where applicable. Italics, Pearson correlation; Non-italics, Spearman correlation. Bold values indicate statistical significance at α = 0.05*.

## Discussion

The overarching goal of the current study was to examine the effects of smoking abstinence on associations between IC and smoking lapse vulnerability. Inhibitory control task performance was positively associated with latency to smoke when collapsing across both sated and abstinent smoking states. Though the main effect of smoking state on IC performance was not significant, abstinence did significantly reduce latency to smoke during the SRT. Furthermore, while IC-Activation in rIFG was found to be associated with smoking sooner on the SRT in the sated condition, abstinence led to increased IC-Activation in insula and dorsolateral prefrontal cortex (i.e., middle frontal gyrus) that was associated with worse IC performance. Middle frontal gyrus activation was also associated with smoking sooner on the SRT.

The findings that smoking abstinence significantly reduced latency to smoke on the SRT concurs with the literature reporting laboratory-based smoking relapse analog tasks (37, 38). However, abstinence was not associated with decreased IC, despite significant self-reports of withdrawal. Recent research points to a particular disruption in sustained attention components of IC tasks (39) rather than a disruption of IC specifically. We did not detect abstinence effects on sustained attention in our behavioral measures (e.g., in Frequent Go trials and Rare Go trials) but posit that MFG recruitment during abstinence may reflect compensation for the attentional demands associated with the IC task above and beyond novelty and stimulus detection, two critical components of sustained attention. Although previous studies have shown detriments in IC due to smoking abstinence (15, 40), many have shown non-significant differences (24, 39, 41, 42). Our results, considered together with the mixed pattern of results in previous studies on the effects of abstinence on IC task performance, reinforce the value of further characterizing the effects of nicotine withdrawal on sustained attention (43–46). Another fruitful avenue for further research may involve studies directly comparing the effects of different durations of abstinence [e.g., (40, 47)].

Results from the current study also reveal that associations between IC and lapse vulnerability are maintained across smoking state. This finding suggests that deficits in IC may represent a smoking endophenotype. Previous research on the role of genetic factors in IC and nicotine addiction further support this idea. For instance, a study in a large cohort of siblings provided evidence that individual variability in IC, as measured by reaction time on a stop-signal task, is significantly heritable (48). A retrospective study in male twins also found that over 50% of variance in smoking cessation success is attributable to genetic factors (49). Conceptualizing IC as an endophenotype with a significant genetic component may also shed light on the relationships between nicotine addiction and heritable comorbid neuropsychiatric disorders, e.g., attention deficit hyperactivity disorder (42). If, indeed, IC is an endophenotype related to nicotine addiction, individual differences in IC may be predictive of cessation success both prior to and after quitting. Future longitudinal research, however, is needed in order to establish whether individual differences in IC and cessation success in smokers are the result of chronic exposure to nicotine or represent pre-disposing factors leading to nicotine dependence.

In the current study, IC task demands reliably activated the rIFG in both smoking state conditions and no significant effects of smoking state on rIFG activation were found. The current study findings are consistent with prior reports in the literature that suggests smoking abstinence significantly impacts executive function broadly and that trait-level deficits in IC may be less sensitive to smoking state, as indicated by recent studies showing no effect of smoking abstinence on rIFG activity during IC and no significant effects on IC behavior (39, 42). However, recent research has reported that abstinence induces relative increases in rIFG response during IC (15). Despite the associations observed in the current study between IC-related rIFG activation and relapse vulnerability under conditions of satiety, this relationship was non-significant in this region under conditions of abstinence. These results, together with our behavioral findings, further bolster the hypothesis of a state-insensitive endophenotype underlying IC and its relationship with smoking relapse vulnerability; however, futher research with a larger sample is needed.

Our *post-hoc* whole-brain exploratory analyses revealed other brain regions in which IC-Activation may be associated with smoking behavior, including the MFG and insula. These findings are consistent with prior work indicating that smoking abstinence leads to a more distributed recruitment of neural resources to perform cognitively-demanding tasks (23, 50), and abstinence disrupts multiple forms of executive function (8, 22, 51). Furthermore, greater IC-Activation in MFG during abstinence was associated with both worse IC task performance and smoking sooner on the SRT. The MFG forms a well-characterized looping circuit with the dorsal striatum, pallidum, and thalamus involved in executive functions (52, 53), including working memory (54) and sustained attention (55). Signaling between MFG and the thalamus is also proposed to mediate top-down control over alertness (56). Compared to non-smokers, sated smokers exhibit hyperactivation in MFG during cognitive control [e.g., (9, 10)]. Building on previous findings demonstrating robust effects of nicotine on sustained attention (11, 57), Kozink et al. showed that smoking abstinence led to decreases in sustained activity in the right MFG over the course of a sustained attention task, but greater transient activity in response to targets (23). These findings by Kozink et al. were interpreted in the context of a “dual mechanisms of control” model which further posits that reactive control processes, in opposition to proactive control, involve transient activation of widespread areas of the PFC and beyond as opposed to sustained activation of the lateral PFC (58). The task used in the current experiment was not optimally suited for probing sustained attention but is designed to assess IC while controlling for novelty detection (one form of attention); therefore we did not explicitly assess sustained activation during the IC task. Our results may, however, suggest that abstinence particularly impairs the proactive control component of the IC task, leading to transient recruitment of additional PFC regions, specifically a region implicated in sustained attention (MFG), in order to meet the prolonged attentional demands of the task. As mentioned previously, this interpretation is consistent with recent findings that abstinence appears to particularly disrupt the components of IC tasks more related to sustained attention (39).

The negative relationship between IC-related insula activation and No Go accuracy may suggest a biasing of attention toward interoception and away from goal-directed behavior during the IC task. Studies point to the insula as a key neural substrate underlying interoceptive monitoring (59) and specifically the interoceptive signals associated with drug craving (60) and withdrawal-induced negative affect (22). Damage to the insula has been shown to disrupt smoking behavior (61) and decrease nicotine withdrawal during abstinence (62). The insula has been shown to play a key role in shifting cognition between the default mode network (DMN) and the executive control network (63). The negative relationship between insula activity and IC we observed may be explained by heightened attention to craving-related internal signals and a subsequent shift toward DMN processing, at the expense of ECN activation. This idea is supported by research indicating that nicotine withdrawal is associated with increased functional connectivity between the insula and the DMN (64) whereas increased functional connectivity between the insula and primary sensory cortices is associated with maintaining abstinence (65).

Although this study provides valuable insight into the relationship between IC and smoking behavior, certain limitations warrant consideration. The smokers in this study were not currently interested in quitting, and further research is needed to assess whether these findings would generalize to treatment-seeking smokers. The laboratory analog for relapse employed herein, while a validated probe for relapse propensity, was limited by not controlling for individual temporal discounting functions, e.g., empirically-derived *k* coefficients reflecting the degree to which the value of a reinforcer is affected by delay in a hyperbolic delay discounting function (66, 67). Because this was not assessed nor controlled for, we could not effectively account for a potential ceiling effect in some participants and/or insufficient monetary reinforcement in others. Future studies which adjust for individual *k* values may resolve additional individual differences. Future directions may also include the use of other laboratory smoking tasks that probe multiple facets of nicotine seeking, e.g., the latency of reaching for smoking paraphernalia [see (68)]. The relatively small sample size for this study also precluded the analysis of sex differences and effects of race and ethnicity, which may be a fruitful direction for future studies.

## Conclusions

In conclusion, these data suggest that individual differences in IC and smoking behavior may reflect an underlying endophenotype that is predictive of relapse vulnerability. Additional translational research examining genotypic factors underlying IC and its relationship with smoking relapse vulnerability are warranted (69). These findings may have applicability to therapies for enhancing IC and ameliorating relapse vulnerability. For example, non-invasive neural stimulation, specifically theta-burst transcranial magnetic stimulation (TMS) to the rIFG, has recently been shown to influence IC among sated smokers (70). Future studies may also explore whether TMS can improve IC in abstinent smokers and impact its relationship with resisting smoking.

## Data Availability Statement

The raw data supporting the conclusions of this article will be made available by the authors, without undue reservation.

## Ethics Statement

The studies involving human participants were reviewed and approved by the Medical University of South Carolina Institutional Review Board. The participants provided their written informed consent to participate in this study.

## Author Contributions

SB analyzed the data, wrote the manuscript, and contributed to the data collection. BF designed the study, supervised the research, and edited the manuscript. Both authors contributed to the article and approved the submitted version.

## Conflict of Interest

The authors declare that the research was conducted in the absence of any commercial or financial relationships that could be construed as a potential conflict of interest.
