# Supplementary material for: Associations Between Smoking Abstinence, Inhibitory Control, and Smoking Behavior: An fMRI Study
Source: Front Psychiatry. 2021 Apr 1;12:592443. doi: 10.3389/fpsyt.2021.592443 (PMC8047070; doi:10.3389/fpsyt.2021.592443)
Supplement: Supplementary file 1 [file Data_Sheet_1.docx]

Supplementary Material

# Supplementary Tables

| Supplementary Table 1. Main Effect of Correct Trial Type on BOLD Activation (*No Go Correct vs. Rare Go Correct)* | | | | | | | | | | | |
| --- | --- | --- | --- | --- | --- | --- | --- | --- | --- | --- | --- |
| *Note*: k_E_ = actual cluster extent; degrees of freedom for model listed next to F in parentheses. | | | | | | | | | | | |
| Abstinent | | | | | | | | | | | |
| Pattern | Hemi-sphere | Region | k_E_ (mm^3^) | | MNI (x,y,z) | | *F*_(1, 50)_*^1^* | | *p* | Mean BOLD^2^ (SE) | |
|  |  |  |  |  |  |  |  |  |  | No Go | Rare Go |
| No Go > Rare Go | Right | IFG | 709 | | 32, 21, -14 | | 45.79 | | <.0001 | 1.74 (0.32) | -0.91 (0.26) |
|  | Right | IFG | 851 | | 50, 8, 26 | | 28.96 | | <.0001 | 1.67 (0.39) | -0.87 (0.31) |
|  | Right | IFG | 64 | | 48, 32, 28 | | 28.54 | | .019 | 1.62 (0.33) | -0.85 (0.35) |
|  | Right | IFG | 84 | | 46, 9, 38 | | 28.08 | | .015 | 1.80 (0.40) | -0.94 (0.37) |
|  | Right | IFG | 47 | | 44, 38, 26 | | 27.28 | | .022 | 1.98 (0.46) | -1.03 (0.40) |
|  | Right | SMA | 419 | | 3, 6, 52 | | 26.08 | | .002 | 1.49 (0.33) | -0.77 (0.33) |
| Rare Go > No Go | *No significant clusters* | | | | | | | | | | |
| Sated | | | | | | | | | | | |
| No Go > Rare Go | Right | IFG | 125 | | 32, 21, -12 | | 30.03 | | .012 | 1.15 (0.25) | -0.78 (0.26) |
|  | Right | IFG | 982 | | 50, 14, -2 | | 27.47 | | <.0001 | 1.42 (0.31) | -0.95 (0.34) |
|  | Right | IFG | 78 | | 45, 10, 34 | | 23.13 | | .017 | 1.29 (0.32) | -0.87 (0.33) |
|  | Right | SMA | 311 | | 12, 6, 62 | | 26.86 | | .003 | 0.98 (0.23) | -0.66 (0.23) |
| Rare Go > No Go | *No significant clusters* | | | | | | | | | | |
| Conjunction | | | | | | | | | | | |
| No Go > Rare Go | Right | IFG | | 125 | | 32, 21, -12 | | 30.03 | .012 | 1.15 (0.25) | -0.77 (0.26) |
|  | Right | IFG | | 219 | | 51, 12, 16 | | 24.03 | .006 | 1.45 (0.34) | -0.97 (0.37) |
|  | Right | IFG | | 44 | | 45, 9, 36 | | 22.08 | .024 | 1.26 (0.33) | -0.85 (0.32) |
| Rare Go > No Go | *No significant clusters* | | | | | | | | | | |
| ^1^ *p* < .05 FWE_voxel_ corrected  ^2^ Mean BOLD from peak voxel 2nd Level adjusted fitted responses | | | | | | | | | | | |

| Supplementary Table 2. Abstinent Condition Whole Brain Analyses: Main Effect of Correct Trial Type on BOLD Activation (*No Go Correct vs. Rare Go Correct)* | | | | | | | | |
| --- | --- | --- | --- | --- | --- | --- | --- | --- |
| *Note*: k_E_ = actual cluster extent; degrees of freedom for model listed next to F in parentheses. | | | | | | | | |
| Abstinent | | | | | | | | |
| Pattern | Hemi-sphere | Region | k_E_ (mm^3^) | MNI (x,y,z) | *F*_(1, 50)_*^1^* | *p* | Mean BOLD^†^ (SE) | |
|  |  |  |  |  |  |  | No Go | Rare Go |
| No Go > Rare Go | Right | Insula | 1461 | 34, 18, -12 | 43.82 | <.0001 | 1.63 (0.30) | -0.91 (0.26) |
|  | Right | Parietal Cortex | 1634 | 52, -40, 54 | 42.48 | <.0001 | 1.50 (0.29) | -0.84 (0.24) |
|  | Right | MFG | 270 | 44, 39, 33 | 39.97 | .001 | 1.55 (0.29) | -0.87 (0.27) |
|  | Left | Putamen | 385 | -20, 9, 2 | 34.68 | <.0001 | 0.83 (0.15) | -0.47 (0.17) |
|  | Right | Parietal Cortex | 159 | 60, -44, 30 | 33.57 | .004 | 1.26 (0.31) | -0.71 (0.18) |
| Rare Go > No Go | *No significant clusters* | | | | | | | |
| Sated | | | | | | | | |
| No Go > Rare Go | Right | Parietal Cortex | 5785 | 38, -40, 39 | 68.86 | <.0001 | 1.13 (0.17) | -0.77 (0.16) |
|  | Right | Insula | 520 | 30, 18, -9 | 40.67 | <.0001 | 1.10 (0.22) | -0.76 (0.20) |
|  | Right | Putamen | 125 | 18, 10, -4 | 34.48 | .005 | 1.00 (0.24) | -0.69 (0.17) |
|  | Left | Insula | 41 | -38, 16, -4 | 32.18 | .017 | 1.39 (0.34) | -0.95 (0.25) |
| Rare Go > No Go | *No significant clusters* | | | | | | | |
| ^1^ *p* < .05 FWE_voxel_ corrected | | | | | | | | |

## Supplementary Figures

**No Go**

Go/Go/No-Go Task

Go


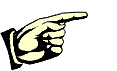

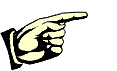


**!**

Rare Go


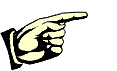


Go


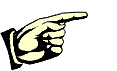


Go


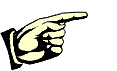


**Supplementary Figure 1.** Experimental Go/Go/No-Go Task. Participants completed the task during fMRI scanning. All stimuli were presented for a 400-msec duration with a 400-msec inter-trial interval over 7.22 minutes.

.
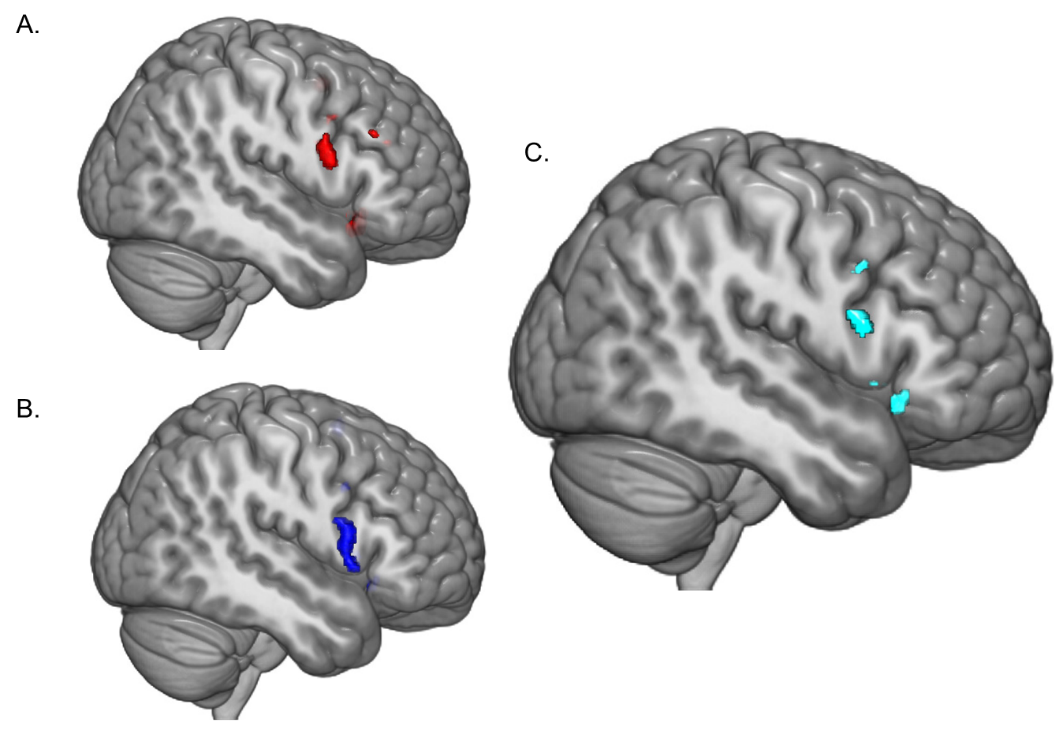


**Supplementary Figure 2. Main Effect of Go/Go/No-Go Task on Brain Activation. (A)** Main effect of Trial Type (Correct No Go, Correct Rare Go) on brain activation under the Abstinent condition. (**B)** Main effect of Correct Trial Type on brain activation under Sated condition. (**C)** Conjunction of main effect of Trial Type on BOLD signal on Sated and Abstinent visits. (Threshold for all models: p<0.05 FWE-corrected).
